# Fu’s subcutaneous needling: a traditional acupuncture radiating new therapeutic potentials

**DOI:** 10.3389/fmed.2026.1846486

**Published:** 2026-07-10

**Authors:** Jinfeng Zhang, Xin Liu, Yan Lu, Binghui Li, Yunjie Cai, Jinlian Liu, Xiaoyong Sun

**Affiliations:** 1 Department of Proctology, Ganzhou Hospital of Traditional Chinese Medicine, Ganzhou, Jiangxi, China; 2 Department of Traditional Chinese Medicine, The First Affiliated Hospital of Gannan Medical University, Ganzhou, Jiangxi, China

**Keywords:** anorectal diseases, Fu’s subcutaneous needling, lower gastrointestinal disorders, pain relief, subcutaneous tissue

## Abstract

**Background:**

Fu’s subcutaneous needling (FSN) is a brainchild of conventional acupuncture and modern medicine. It is initially used to mask pain in musculoskeletal conditions by directly stimulating the subcutaneous tissue using a special trocar needle. Currently, the modern clinical indications for FSN are gradually expanding.

**Methods:**

Through literature review of FSN in treating pain and non-pain conditions published from 1^st^ January 2020 to 31^st^ December 2025 in both Chinese and English databases, including the CNKI, Wanfang, PubMed, and Cochrane, this article thoroughly described the evolution of FSN from the 1.0 (soft tissue pain) and 2.0 (postoperative pain, neuralgia pain, cancer pain) eras to the 3.0 era (lower gastrointestinal disorders) in clinical settings. Evidence from case reports, clinical trials and animal experiments was summarized.

**Results:**

FSN is initially created to relieve pain conditions through the core mechanisms of tightened muscle and reperfusion approach. It is now radiated into the treatment of non-pain conditions like lower gastrointestinal disorders by indirectly manipulating a neuro-endocrine-immune network, possessing high efficacy and safety profile.

**Conclusion:**

This review provides new insights into FSN in alleviating pain and non-pain conditions, especially in treating lower gastrointestinal and/or anorectal diseases.

## 1 Introduction

### 1.1 History of FSN

Fu’s subcutaneous needling (FSN) is a novel type of acupuncture invented in 1996 by the famous acupuncture scientist Professor Fu Zhonghua (1). Rather than an accidental invention, FSN is a mature fruitage growing from an integration of conventional acupuncture and modern medical anatomy, physiology and pathology. Conventional acupuncture is long-term limited by a weaker efficacy in treating soft tissue pain and dysfunction (2). The subcutaneous layer, consisting of loose connective tissue and large blood vessels and nerves, allows the Fu needle to reach the target easily, thus bringing with a more favorable therapeutic efficacy. This serves as the cornerstone of FSN in the initial clinical application to relieve soft tissue pain. The development of FSN has gone through several important stages. During the initial stage (1.0 era), Professor Fu gradually established and perfected the theoretical bases of FSN, including the theories of tightened muscle and reperfusion approach (3). FSN, in the 1.0 era, is mainly applied to treat musculoskeletal system diseases (4). With the maturation and popularization of FSN in clinical practice, the scope of indications for FSN has continuously expanded during the developmental stage (2.0 era), including postoperative pain, neuralgia pain and cancer pain. In the 21st century (3.0 era), a growing number of case reports, clinical trials and animal studies on FSN emerged. It not only relieves pain, but also manages non-pain conditions in the lower gastrointestinal tract like irritable bowel syndrome, constipation and anorectal diseases.

### 1.2 Unique characteristics of FSN

**Figure 1:**
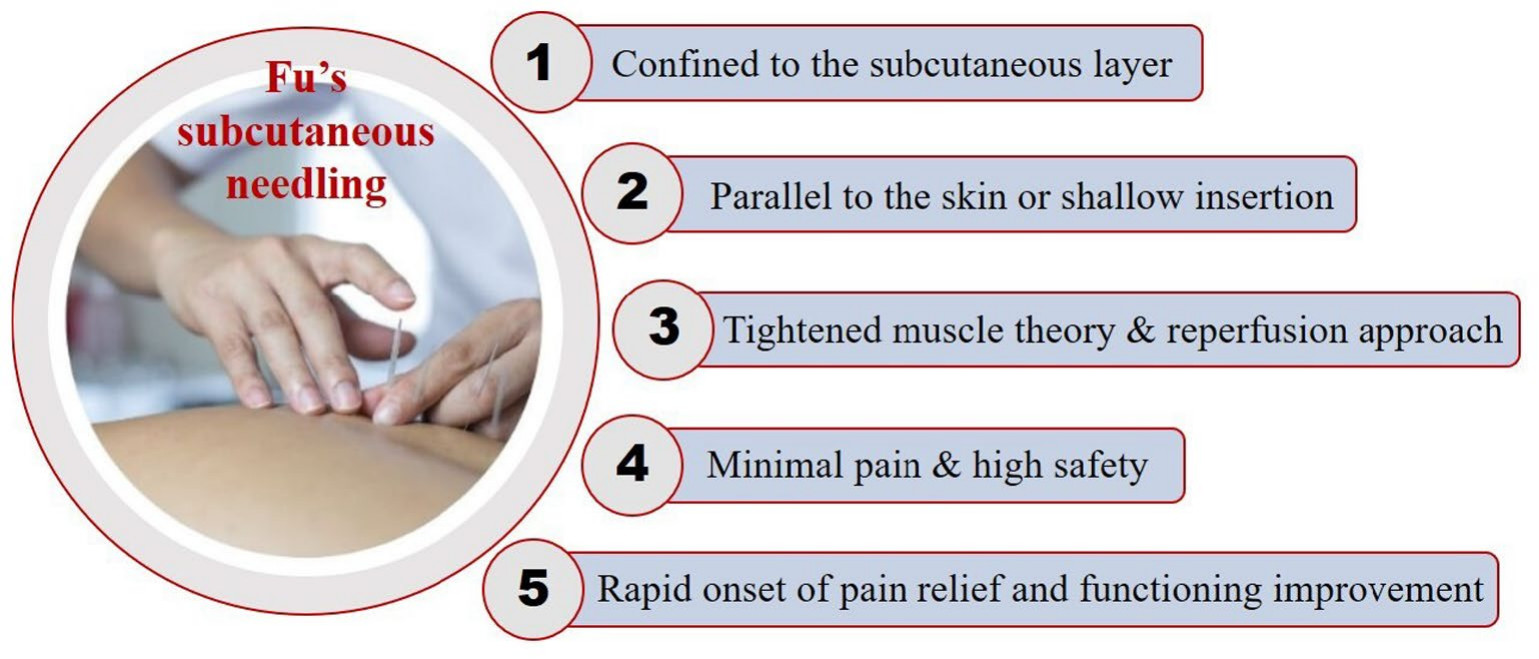
Unique characteristics of FSN.

FSN possesses unique characteristics compared to conventional needling therapies (Table 1). First, FSN is confined to the subcutaneous layer enriched in blood vessels, lymphatic vessels, nerve endings and various cellular components, where the metabolic activities and pain signal transduction are abundant. Second, the insertion direction and depth of FSN are divergent from the conventional acupuncture. FSN needles are inserted parallel to the skin or at a shallow angle, until reaching the subcutaneous layer, but not the muscle layer. Such a precisely controlled procedure limited in the subcutaneous layer requires a thorough grounding in anatomical knowledge and experienced operating skills. Third, the theories of tightened muscle and reperfusion approach are core mechanisms responsible for the action of FSN (5). It improves local blood circulation and lymphatic circulation through needle stimulation, thus promoting the removal of metabolic waste and the supply of nutrients. Fourth, the shallow insertion of FSN needles minimizes pain and ensures the high safety, without damaging deep tissues and important organs. Fifty, FSN is established for its rapid effect on pain relief and functioning (6). It addresses myofascial trigger points (MTrPs), allowing blood flow perfusion and muscle relaxation (Figure 1). Interestingly, FSN is characterized by a strict limitation of needle insertion into the subcutaneous layer, without penetrating muscles or deep tissues. Acting on the myofascial trigger points (MTrPs), this superficial approach effectively alleviates muscle pain and spasm by mediating the fascia-nerve interface, where local microcirculation in the fascia and electrical neural signals that influence muscle function are indirectly activated.

**Table 1:** Characteristics of FSN and conventional acupuncture.

Characteristic	**FSN**	**Conventional needling**
Layer	Subcutaneous layer	Muscle layer
Manipulation	Fan-shaped swaying motion	Vertical insertion, and lifting-thrusting and twisting-rotating needle manipulations
Mechanism	Relaxation of subcutaneous connective tissue and improvement of local microcirculation	Regulation of meridians, Qi and blood; Regulation of Yin and Yang; Strengthening body resistance and eliminating evil
Indications	Soft tissue pain, neuralgia pain, cancer pain, lower gastrointestinal disorders, anorectal diseases	Systemic diseases
Needles	Trocar needles consisting of soft tube, protecting tube and needle core	Stainless steel needles
Adverse events	Minimal adverse events	Mild pain

FSN, Fu’s subcutaneous needling.

### 1.3 Search strategy

In the present review, we systematically searched articles reporting the clinical application of FSN published from 1st January 2020 to 31st December 2025 in both Chinese and English databases, including the CNKI, Wanfang, PubMed, and Cochrane. Keywords for searching qualified articles consisted of Fu’s subcutaneous needling, FSN, pain, lower gastrointestinal disorders, and non-pain management. Though a PRISMA flow diagram was not provided, the process of literature search was strictly structured to ensure the reproducibility and academic rigor.

## 2 Physiological basis of FSN

### 2.1 The theory of tightened muscle

The theory of tightened muscle, as the core mechanism of FSN, holds that an abnormal functioning of tightened muscles induces pathological changes (7). From the perspective of muscle physiology, muscles work relying on accurate neural innervation, sufficient blood supply, and adequate metabolism. Pathological changes in muscles, such as muscle tension, spasms and ischemia, not only influence muscle functioning, but also affect other systems and organs via the neuroreflex mechanism. From the perspective of neurophysiology, the impairment of the cerebellum and basal ganglia, as brain regions dominating coordination and control of movement, is involved in muscle dysfunction.

### 2.2 Reperfusion approach

Reperfusion is an important procedure of FSN by active movements of patients in the affected area while the needle is in place. A combination of FSN and reperfusion benefits the release of MTrPs, achieving the goal of muscle relaxation, pain relief, and management of non-pain conditions.

The reperfusion approach, as the other critical mechanism of FSN, aims to smooth local blood flow and lymphatic circulation by FSN (8). First, mechanical stimulation via FSN directly acts on the blood vessel wall, leading to contraction or relaxation of vascular smooth muscle. Second, FSN activates vascular endothelial cells and promotes the release of vasoactive substances, such as nitric oxide and prostacyclin (9). Third, FSN alters the neuromodulation of blood vessels through the neuroreflex mechanism, including the sympathetic and parasympathetic nerves. Fourth, the contraction of the lymphatic vessels can be enhanced by FSN, thus promoting the lymph fluid flow and maintaining the normal metabolism. Fifth, the release of inflammatory factors, activities of inflammatory cells and pro-inflammatory mechanisms can be triggered by FSN, serving as critical events involved in the reperfusion (3).

Previous work has focused on the exploration of FSN in relieving the musculoskeletal system diseases and the underlying mechanisms based on the two core theories of FSN. With a clear illustration of the tightened muscle and reperfusion explaining the efficacy of FSN in relieving soft tissue pain, increasing studies have investigated the potential of FSN in managing other types of pain and even non-pain conditions, aiming to broaden its clinical application. Within this physiological framework, FSN goes beyond the tradition of relieving soft tissue pain to the new treatments of postoperative pain, neuralgia pain, and non-pain conditions like lower gastrointestinal disorders and/or anorectal diseases.

## 3 Clinical application of FSN

### 3.1 Low back pain

Previous studies have proven the immediate effect of FSN on masking low back pain. The pathogenesis of low back pain is complicated, involving multiple anatomical structures like muscles, ligaments, intervertebral discs, and small joints (10, 11). The theory of tightened muscle lays a foundation for the efficacy of FSN in treating low back pain. Notably, FSN procedures should be modified based on individual conditions due to multiple layers of muscles in the waist and back, and varying thickness of subcutaneous fat. For example, a shallow insertion is essential when the needle penetrates into the thin layer of subcutaneous tissue next to the spinous process of the lumbar vertebra. A longer needle is needed for treating pain within the area of the quadratus lumborum beneath thick subcutaneous tissue.

Fu et al. reported a cohort of patients with chronic benign back pain between the 12th rib and the gluteal fold treated with FSN using the catheter IV to replace the FSN needles, thus ensuring a high repeatability (6). Briefly, the catheter IV obliquely penetrates into the insertion point at the same side of the suffered back, advances parallel to the skin to the subcutaneous layer, retracts 3 ms to make the tip wrapped in the soft tube, and smoothly and rhythmically fan-shaped sways for 200 times within 2 min. Finally, FSN greatly relieves motion-related pain and that under pressure. Ma et al. emphasized the importance of determining tightened muscles in patients with chronic non-specific low back pain prior to FSN. Similarly, FSN effectively alleviates pain, and improves functioning and quality of life for a long period of 12 months (12). Zhang et al. (13) demonstrated that FSN for 3 consecutive days (200 times of fan-shaped swaying regularly within 2 min plus 10-s reperfusion of actively or passively relaxing the tightened muscle) significantly decreased scores on the Visual Analogue Scale (VAS) and the Fear Avoidance Beliefs Questionnaire (FABQ) at each follow-up visit in patients suffering from chronic non-specific low back pain.

### 3.2 Soft tissue pain of knee osteoarthritis (KOA)

KOA, as a common condition in the elderly, is a painful disease derived from the joint itself and surrounding soft tissues of muscle groups (e.g., quadriceps, hamstring, triceps, and tibial anterior muscles). FSN is found to alleviate pain and enhance the range of motion in KOA patients through enhancing the function and blood supply of soft tissues around the knee joint (14).

Mou et al. (15) demonstrated the efficacy of FSN in improving joint function of patients with mild-to-moderate KOA. Inserted 3–5 cm away from the periphery of the tightened muscle, FSN acts on MTrPs of knee osteoarthritis and sways at a frequency of 100 beats/min for 2 min. The following reperfusion approach, consisting of tightened muscle extension and/or resistance movements, is performed for two sessions, each lasting 10 s. A combination of swaying movements and reperfusion of tightened muscles largely increases the quality of life in KOA patients. A randomized, single-blinded study similarly reported the long-lasting efficacy of FSN in treating degenerative KOA (16). Briefly, patients are managed by FSN via inserting toward the patella and swaying 45 movements within 30 s, followed by the reperfusion approach via 1-min cycles of foot dorsiflexion and knee flexion and extension. Chiu et al. (17) pinpointed the insertion site of an FSN needle in the upper third of the line connecting the superior margin of the patella and the anterior superior iliac spine line. Combined with the reperfusion approach, FSN effectively relieves soft tissue pain around the knee on osteoarthritis. Considering the thin level of subcutaneous tissue, the vastus lateralis of quadriceps femoris is selected as the tightened muscle to be intervened by four sessions of FSN for treating early-to-mid stage KOA. Each session consists of 20 s of swaying movements plus 10 s of reperfusion for 3 replicates. This management greatly enhances blood flow and oxygenation in the affected knee joint, without leading to severe adverse events (18). Liu et al. (19) specifically quantified the insertion depth of FSN needles in the treatment of KOA, which are first inserted 5 mm toward the tightened muscle, and then advanced subcutaneously for 25–35 mm.

### 3.3 Shoulder and neck pain

Shoulder and neck pain is diversely manifested as muscle tension, cervical spondylosis, scapulohumeral periarthritis, etc. The shoulder and neck muscles are arranged in a complex multi-level structure, broadly categorized into the superficial layer (e.g., trapezius and sternocleidomastoid) for gross movement and the deep layer (e.g., levator scapulae and deep neck muscles) for fine movement and posture maintenance. FSN, via a direct act on the tightened trapezius, alleviates pain through improving the circulation (4). Notably, if acting on the sternocleidomastoid, the procedure of FSN should be strict, due to the presence of important vessels and nerves situated in this area.

An FSN needle is inserted in the center of the extensor muscle on the affected side and swayed 50 times within 30 s, followed by 3 cycles of the upper trapezius contraction within 2 min, known as the reperfusion approach. As a result, patients with chronic neck pain remarkably benefit from FSN with an immediate outcome of pain relief (7). Ultrasonic elastography confirms the role of FSN in reducing the thickness and elasticity of the upper trapezius in individuals suffering from shoulder and neck pain (20). A retrospective case-matched comparative study suggested an insertion depth of 4 mm into the shoulder area with the most severe pain, a rhythmical sway for 50–100 times within 2 min, and a reperfusion approach for all-plane movements of the shoulder joint; this treatment alleviates the pain and increases the range of motion obviously (21). A cohort of 120 adult male patients with unilateral or bilateral shoulder and neck pain is managed by FSN with needles inserted at 6–10 cm inferior to the tightened muscle. FSN needles remain subcutaneous for 24 h in individuals with severe or long-lasting pain. During the follow-up period of 1 month to 2 years, FSN achieves an overall effective rate of 98% in relieving shoulder and neck pain (22).

### 3.4 Postoperative pain

Stepping into the 2.0 era of FSN, its range of clinical indications has expanded from pain-related musculoskeletal disorders into postoperative pain, neuralgia pain, and cancer pain. Postoperative pain troubles the vast majority of surgically treated patients, leading to reduced quality of life and delayed rehabilitation. Pain medications, although serving as the primary analgesic approach, bring adverse events and the risk of addiction. FSN is a non-pharmacological treatment with unique advantages in the postoperative pain control. First of all, FSN, following the gate control therapy, inhibits the conduction of pain signals through neuromodulation (23). Second, FSN contributes to wound healing and anti-inflammation via improving the blood flow in the surgical site (12). Third, FSN eases muscle tension postoperatively, especially the compensatory muscle tension (24).

Wu et al. (24) described the application of FSN to postoperative patients with degenerative lumbar spinal disorders. Briefly, FSN needles are inserted into the subcutaneous layer in the inferior margin of the scapula and posterior superior iliac spine on bilateral sides. Under the guidance of ultrasonography, needles are retracted and regularly swayed for 200 times within 2 min. The reperfusion approach, via 3 repeated cycles of 10-s hip extensions and 10-s rests, is performed on each side. Finally, patients demonstrate pain relief and less muscle hardness immediately at 1 h postoperatively. A cohort of 64 patients with osteoporotic vertebral compression fracture (OVCF) after percutaneous vertebroplasty (PVP) are intervened with 8 cycles of FSN. In each cycle, FSN is performed daily for 6 days, with a frequency of 60 sways per minute for 3 min. Surprisingly, FSN effectively relieves postoperative pain of OVCF and reducing relative levels of neuromodulators associated with pain perception (neuropeptide Y, 5-HT, and substance P) (25). Cheng et al. (26) consistently proved the efficacy of 3 sessions of FSN, acting on the subcutaneous layer 3–4 cm away from the tightened muscles once daily, in lowering the VAS score and increasing the Japanese Orthopaedic Association (JOA) score in postoperative OVCF patients. However, reperfusion exercises are not given to PVP-treated patients, potentially resulting in a less favorable analgesic outcome. Long et al. particularly performed FSN in elderly OVCF patients (60–89 years) following PVP who suffered from residual low back pain. A total of 150–200 fanning, sway-like movements of FSN needles combined with the reperfusion approach (lifting the affected leg with resistance) significantly decreases the VAS score from 6 points at baseline to 1 point at 3 months postoperatively. It suggests that FSN is a gratifying alternative to reduce the use of analgesic drugs, especially to older adults or individuals with poor physical conditions (27).

### 3.5 Neuralgia pain

Neuralgia, as a special type of pain radiating from nerves, challenges the conventional medication therapy management (MTM). At present, clinical evidence shows encouraging outcomes of FSN in relieving postherpetic neuralgia (PHN), trigeminal neuralgia, and sciatica. Guo et al. reported the extremely high effective rate of FSN combined with laser needle knife treatment in PHN, compared with the monotherapy of electroacupuncture (94.1% vs. 68.6%), although the reperfusion approach is not given (28). FSN lowers hyperalgesia in PHN patients via the converse piezoelectric effect of neutralizing the pain signal. Due to rhythmical and swaying movements of FSN needles, action potentials transmitted along the sarcolemma and sarcoplasmic reticulum are blocked. Consequently, the transmission of neurokinin substances is interrupted by inhibiting axon sprout and reducing the sensitivity of skin chemoreceptors. Additionally, the reperfusion approach acts on PHN-associated MTrPs accelerates the flow of blood and Qi, and thus reduces pain. The retaining of FSN needles in the subcutaneous layer berates the gathering of poison and evil. All these measures synergistically achieve the goal of pain relief (29). FSN, applying on the MTrPs within the neck and face regions at a frequency of 200 rhythmical swaying movements within 2 min, offers an immediate and complete pain relief to patients with trigeminal neuralgia (30). In a rat model of sciatica, fan-shaped sways of FSN needles for 100 times in 1 min on day 1 plus the daily reperfusion approach on day 1, 3, 5 and 7 significantly increase the mechanical pain threshold and improve the mitochondrial structural damage and dysfunction (31). However, the outcome of FSN in treating sciatica needs a further validation in clinical trials.

### 3.6 Cancer pain

Cancer pain, as a common but miserable symptom in cancer patients, severely reduces the quality of life and treatment compliance. Overall, 44.5% of cancer survivors suffer from cancer pain, which is moderate-to-severe in 30.6% of them (32). Medications, as the mainstream treatment of cancer pain, are limited in clinical practice due to adverse events. FSN offers a non-pharmacological option for a monotherapy or a combination therapy against cancer pain, without leading to side effects like nausea, vomiting, and respiratory depression.

Chen et al. (33) explored the feasibility of FSN combined with oxycodone hydrochloride in masking moderate-to-severe cancer pain. Large-scale, fan-shaped, swaying movements of FSN needles for 5 min daily and needle retention in the subcutaneous layer for 30 min are performed for 14 days. Finally, FSN significantly decreases the Numerical Rating Scale (NRS) and EORTC Core Quality of Life questionnaire (EORTC QLQ-C30) scores, suggesting a satisfactory pain relief. Besides the direct analgesic effect, FSN indirectly reduces anxiety and improves the quality of life in cancer patients. Mechanically, FSN at a swaying movement frequency of 60 repeats/min for 3 min significantly decreases serum 5-HT, PGE2, HIF-1 and VEGF levels in advanced liver cancer patients with the traditional Chinese medicine (TCM) syndrome type of Qi stagnation and blood stasis. It indicates the potential role of FSN in inhibiting pain signal transduction and angiogenesis during cancer growth. A combination of daily FSN on the Ashi Acupoint and morphine sulfate (30 mg, b.i.d.) for 14 days not only reduces the dosage of analgesics, but also improves the quality of life in cancer patients with moderate pain (34). Liu et al. (35) consistently reported clinical benefits of six sessions of FSN and reperfusion approach to individuals suffering from cancer pain, manifesting as significantly reduced Hamilton Depression Scale (HAMD) score and Belief Pain Index (BPI).

### 3.7 Lower gastrointestinal disorders

**Figure 2:**
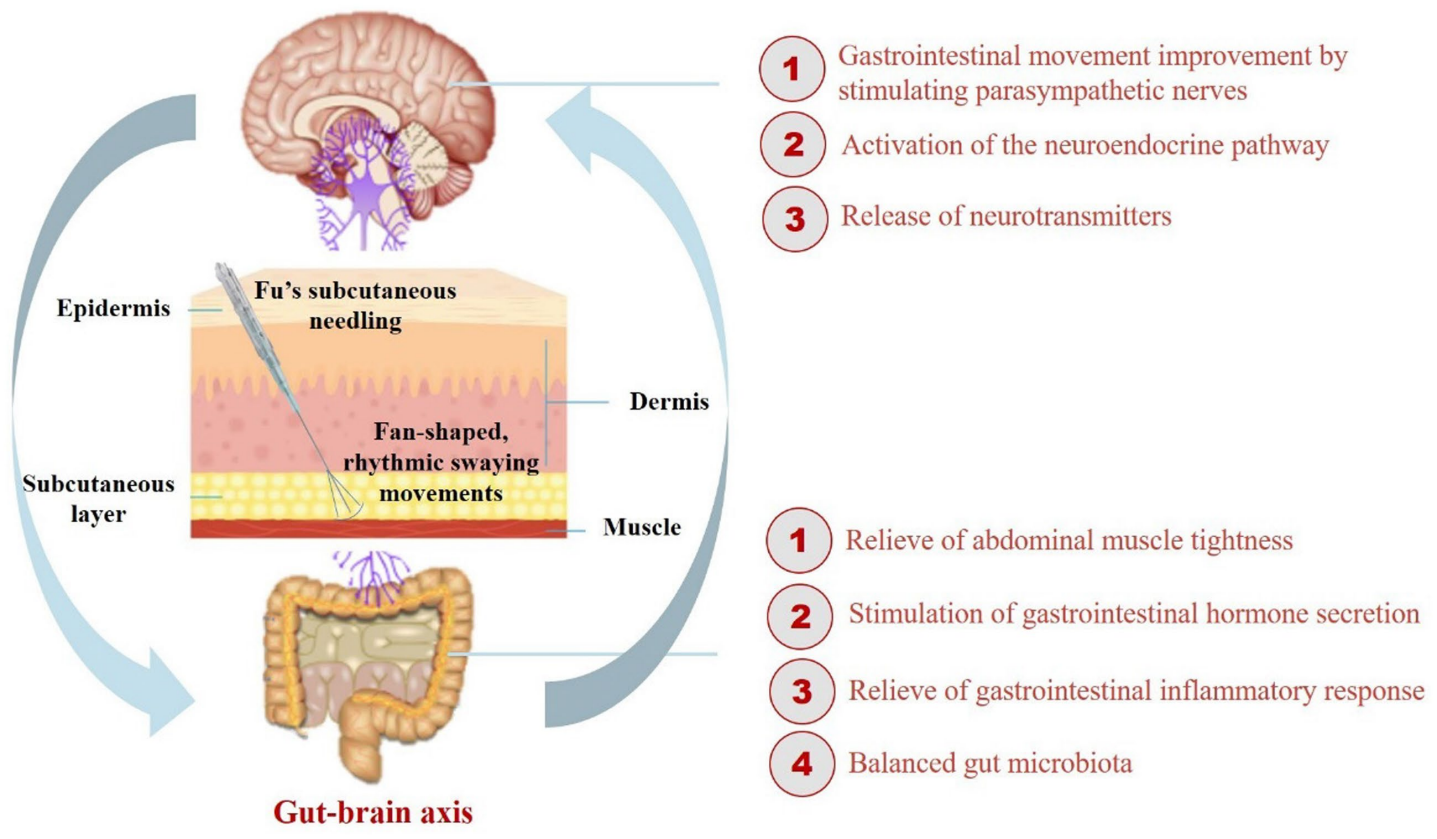
FSN alters the gut-brain axis by directly acting on tightened abdominal muscles.

In the 3.0 era, the clinical application of FSN is no longer limited to pain-related diseases, but tailoring tricky, non-pain conditions in the lower gastrointestinal tract. Patients with lower gastrointestinal disorders generally suffer from abdominal muscle tension, spasms or dysfunction. The tightening of muscles eventually disrupts the regulation of abdominal pressure, bowel movement and emptying of the gastrointestinal tract, and even the inner-balance in gut microbiota. FSN, through directly acting on tightened abdominal muscles, alters the complicated interplay of the brain and gut, as well as relevant nerves and gut microbiome involved in the gut-brain axis (Figure 2). Changes in the abdominal microenvironment following FSN and reperfusion approach provide a feedback loop to the brain. Then, abundant neurotransmitters are released to restore normal gastrointestinal movements, visceral sensitivity, immune function, pro-inflammatory and inflammatory balance, and gut microbiota. We therefore proposed that FSN relieves pain and non-pain conditions through a common mechanism. Generally, tightened muscle and reperfusion approach are the fundamental mechanisms of FSN in improving the well-being. Indirectly, it stimulates subcutaneous nerve endings to release endorphins and other analgesic substances, thereby inhibiting pain transmission. Meanwhile, FSN modulates non-pain conditions via a neuro-endocrine-immune network, benchmarking its therapeutic efficacy against lower gastrointestinal disorders. However, the heterogeneity of FSN in clinical benefits should not be ignored.

A growing number of clinical evidence has supported the efficacy and safety of FSN in treating irritable bowel syndrome (IBS) (36–40), constipation (41–52), and anorectal diseases (53–58). Li et al. (36) compared the clinical outcomes of patients with mild-to-moderate irritable bowel syndrome with diarrhea (IBS-D) managed by compound glutamin versus compound glutamin plus FSN. After 14 days of medications and FSN once every other day, FSN provides additional benefits, as shown by increased serum IFN-*γ*, increased abundance of *Enterobacter*, and decreased abundance of *Bifidobacterium*. Moreover, FSN effectively alleviates clinical symptoms, reduces the visceral sensitivity and improves rectal compliance in IBS-D patients (36). Abundant evidence shows that FSN brings favorable changes in the gut microbiota among IBS patients, which could be attributed to mediatory effects on the parasympathetic nervous system, secretion of gastrointestinal hormones, and immune system function (37, 39). Through literature review, we provided a summary of articles having reported FSN in treating IBS in Table 2.

**Table 2:** A summary of clinical application of FSN to IBS.

Disease	Study design and demographics	Interventions	FSN procedures	Major findings	Refs.
Mild-to-moderate IBS-D	Sample size: 28; Age: 49.5 ± 15.1 yrs.; Sex ratio: 17:13.	FSN plus glutamin entersoluble capsules	Frequency: once every other day, for 14 days; Procedures: swaying movements plus reperfusion approach.	1. Decreased abundance of *Bifidobacterium*, and increased abundance of *Enterobacter* (*p* < 0.05); 2. Increased serum IFN-γ (*p* < 0.05); 3. No adverse events.	(36)
IBS	Sample size: 56; Age: 40.23 ± 4.31 (22–57) yrs.; Sex ratio: 5:9; Course of disease: 5.04 ± 0.57 (1–9) yrs.	FSN plus pinaverium bromide and glutamin entersoluble capsules	Frequency: once every other day for 4 weeks.	1. Increased clinical efficacy (96.43% vs. 83.93%, *p* < 0.05) 2. Shortened symptom regression of abdominal pain, bloating and diarrhea (*p* < 0.05) 3. Increased defecation, pain and sensory thresholds (*p* < 0.05) 4. Increased abundances of *Bifidobacterium*, *Lactobacillus*, *Enterobacter* and *Bacteroides* (*p* < 0.05) 5. Decreased vasoactive intestinal peptide, and increased gastric motility and substance P level (*p* < 0.05).	(37)
IBS-D	Sample size: 33 Age: 37.70 ± 11.51 yrs. Sex ratio: 5:6 Course of disease: 12.00 (9.50, 36.00) months Primary outcomes: VSI score Secondary outcomes: Rectal capacity and sensory thresholds, IBS-SSS, IBS-QOL.	FSN	Frequency: twice per week for 6 weeks; Procedures: Two cycles of 50–100 swaying movements within 1 min on Tianshu, Daheng, Jimen, and Shangjuxu Acupoints plus 1-min reperfusion approach, with an interval of 10 min.	1. Decreased VSI and IBS-SSS score at 3 and 6 weeks of treatment (*p* < 0.05), and increased IBS-QOL score (*p* < 0.01) 2. Increased sensory threshold (*p* < 0.01) 3. Increased clinical efficacy (90.90% vs. 82.35%, *p* < 0.05) 4. Two cases of mild subcutaneous hematoma.	(38)
IBS-D	Sample size: 46 Age: 50.14 ± 11.64 (18–70) yrs. Sex ratio: 12:11 Course of disease: 4.11 ± 1.21 (0.5–8.0) yrs.	FSN plus umbilical moxibustion with herbal medicine	Frequency: once daily for 5 days and an interval of 2-day off, for 4 cycles Procedures: swaying movements plus reperfusion approach.	1. Decreased TCM syndrome and IBS-SSS scores (*p* < 0.05) 2. Increased clinical efficacy (93.48% vs. 78.26%, *p* < 0.05) 3. Decreased motilin, vasoactive intestinal peptide and somatostatin (*p* < 0.05) 4. Increased defecation, pain and sensory thresholds (*p* < 0.05) 5. Increased abundances of *Bifidobacterium*, *Lactobacillus* and *Bacteroidetes*, and decreased abundance of *Enterobacter* (*p* < 0.05).	(39)
IBS-D with the mixed cold and heat syndrome	Sample size: 30 Age: 36.07 ± 10.59 yrs. Sex ratio: 13:17 Course of disease: 35.23 ± 20.42 months.	FSN plus Wumei Pills	Frequency: once every other day for 4 weeks Procedures: 600–100 swaying movements per minute for 10 min on Zhongwan and Tianshu Acupoints plus reperfusion approach.	1. Decreased total TCM syndrome score (*p* < 0.05) 2. Increased IBS-SSS scores, except for the score of satisfactory with bowel habits 3. Decreased VSI score 4. Increased defecation and pain thresholds 5. Low recurrence (6.9% vs. 32%) and high safety profile (one case of mild-to-moderate subcutaneous hematoma in the treatment group and cured).	(40)

FSN, Fu’s subcutaneous needling; IBS, irritable bowel syndrome; IBS-D, irritable bowel syndrome with diarrhea; TCM, traditional Chinese medicine; IBS-SSS, Irritable Bowel Syndrome Severity Scoring System; VSI, visceral sensitivity index; IBS-QOL, Irritable Bowel Syndrome Quality of Life.

TCM theory advocates that myopathy, or muscle weakness, is a risk factor for decreased bowel movements and constipation. Through rhythmic, high-amplitude contractions of muscle tissues within the colon and rectum walls, peristalsis is stimulated to accelerate the digestion and absorption of food residues in the intestines and push them to the anus, ultimately achieving the purpose of defecation. FSN is an exquisite art to target the tightened muscles of the lower abdomen, thus facilitating defecation. Jiang et al. demonstrated that FSN combined with modified Zhizhu Decoction greatly increases complete spontaneous bowel movements (CSBMs) per week in patients with slow transmission constipation (STC). Mechanically, FSN stimulates gut motility by upregulating substance P and motilin, and reduces neurotransmitters (vasoactive intestinal peptide and nitric oxide) that are responsible for relaxing the gastrointestinal system (42). In addition to the immediate effect of stimulating bowel movements and shortening the interval of defecation, FSN also rescues constipation-induced poor quality of life and relieves negative emotions. Clinical applications of FSN to constipation patients are summarized in Table 3.

**Table 3:** A summary of clinical applications of FSN to constipation.

Disease	Demographics	Interventions	FSN procedures	Major findings	Refs.
STC with the syndrome of spleen deficiency and Qi stagnation	Sample size: 50 Age: 43.00 ± 7.90 yrs. Sex ratio: 11:14 Course of disease: 8.29 ± 2.27 yrs. Duration of defecation: 31.36 ± 2.05 min Interval of defecation: 6.10 ± 1.88 days.	FSN plus modified Zhizhu Decoction	Frequency: once daily for 4 weeks Procedures: 200 swaying movements within 2 min plus reperfusion approach	1. Increased clinical efficacy (94.0% vs. 82.0% at 1 month, and 88.0% vs. 76.0% at 6 months, *p* < 0.05) 2. Decreased scores of the constipation symptoms, constipation quality of life, and TCM syndrome score (*p* < 0.05) 3. Increased CSBMs per week (*p* < 0.05) 4. Increased substance P and motilin, and decreased vasoactive intestinal peptide and nitric oxide (*p* < 0.05).	(42)
STC with the syndrome of Qi stagnation	Sample size: 30 Age: 59.92 ± 3.61 (30–69) yrs. Sex ratio: 2:3 Course of disease: 3.81 ± 0.55 (1–6) yrs.	FSN plus biofeedback therapy	Frequency: 3 times per week for 1 month Procedures: 3 swaying movements with 15 s per time plus 5 min local compression (reperfusion approach).	1. Increased clinical efficacy (*p* < 0.05) 2. Decreased TCM syndrome and PAC-QOL scores (*p* < 0.05) 3. Decreased anal basal pressure, maximum systolic pressure of anal canal, and threshold of defecation sensation (*p* < 0.05).	(44)
Chronic STC	Sample size: 35 Age: 41.07 ± 4.98 (34–62) yrs. Course of disease: 4.42 ± 1.22 (2–9) yrs.	FSN plus biofeedback therapy and pulucapride succinate	Frequency: 5 times per week for 4 weeks Procedures: 200 swaying movements within 2 min.	1. Increased clinical efficacy (*p* < 0.05) 2. Increased BSFS scores, and decreased SCL-90 and PAC-QOL scores (*p* < 0.05) 3. Decreased right, left and total colonic transit time (*p* < 0.05) 4. Increased 3-s contraction, 10-s contraction and 10-s relaxation pressure of the external anal sphincter (*p* < 0.05) 5. Increased 5-HT, and decreased somatostatin and vasoactive intestinal peptide levels (*p* < 0.05).	(43)
FC	Sample size: 53 Age: 67.9 ± 9.0 (43–82) yrs. Sex ratio: 14:39 Course of disease: 16.31 ± 8.16 (1–25) yrs.	FSN plus *Bifidobacterium* triple viable capsules	Frequency: once daily for the first 3 days, and once every other day for 3 times Procedures: 50 fan-shaped swaying movements within 30 s plus reperfusion approach.	1. Increased clinical efficacy (92.16% vs. 90.56%) 2. Increased BSFS and GQLI-74 scores (*p* < 0.05) 3. Increased motilin and gastrin levels, and decreased vasoactive intestinal peptide levels (*p* < 0.05) 4. Mild adverse events, including 2 cases of headache and dizziness, 1 case of frequent micturition, and 2 cases of nausea and vomiting (adverse event rate: 7.84% vs. 28.30%).	(52)
FC	Sample size: 27 Age: 59.1 ± 4.6 (34–80) yrs. Sex ratio: 11:19 Course of disease: 8.6 ± 2.1 (0.5–20) yrs.	FSN plus pelvic biofeedback therapy	Frequency: once every 3 days for 5 times Procedures: 2–3 swaying movements with 15 s per time.	1. Increased clinical efficacy over time (overall efficacy: 96.30%; efficacy on day 5, 9, 13, 17, and 20: 55.56, 66.67, 88.89, 96.30, and 96.30%, respectively) 2. Decreased scores of primary and secondary symptoms of constipation at day 5, 9, 13, 17 and 20 (*p* < 0.05).	(45)
Chronic FC	Sample size: 40 Age: 46.2 ± 6.2 (18–85) yrs. Sex ratio: 7:3 Course of disease: 15.0 ± 6.3 (0.5–20) yrs.	FSN plus self-designed laxative prescription	Frequency: 2–3 times per week for 2 weeks Procedures: swaying movements plus 2–3 repeats of 10-s reperfusion, with an interval of 10 s.	1. Increased clinical efficacy (*p* < 0.05) 2. Decreased scores of the interval between bowel movements, nature of bowel movements, and difficulty in defecation (*p* < 0.05) 3. Improved quality of life (*p* < 0.05).	(41)
FC	Sample size: 40 Age: 44.3 ± 12.9 (19–70) yrs. Sex ratio: 21:19 Course of disease: 7.5 ± 2.1 months.	FSN plus *Bifidobacterium* quadruple viable capsules and mosapride	Frequency: once daily for a month Procedures: 200 swaying movements within 2 min on major acupoints (Tianshu, Zhigou, Shuidao, Guilai Acupoints) and adjunct acupoints (Hegu and Neiting Acupoints for intestinal heat; Taichong and Zhongwan Acupoints for intestinal Qi stagnation; Piyu and Qihai Acupoints for spleen deficiency and Qi weakness; Zusanli and Sanyinjiao Acupoints for blood vacuity and fluid deficiency) plus reperfusion approach.	1. Increased clinical efficacy (*p* < 0.05) 2. Increased defecation times per week, improved stool form, and alleviated difficulty in defecation (*p* < 0.05).	(46)
FC	Sample size: 30 Age: 63.58 ± 8.95 (32–75) yrs. Sex ratio: 3:2 Course of disease: 23.70 ± 21.12 (6–48) months.	FSN	Frequency: once every other day, three times per week, for 2 weeks Procedures: 200 swaying movements within 2 min plus reperfusion approach.	1. Increased clinical efficacy (*p* < 0.05) 2. Decreased PAC-QOL, physiological discomfort, social physiological discomfort and anxiety scores (*p* < 0.05) 3. Improved satisfaction (*p* < 0.05).	(49)
Post-stroke constipation	Sample size: 40 Age: 57.94 ± 3.21 (36–79) yrs. Sex ratio: 23:17 Course of stroke: 20.74 ± 4.64 (2–39) months Course of constipation: 5.43 ± 4.30 (3–27) days.	FSN plus rhubarb powder acupoint application	Frequency: once day for the first 3 days, and once every other day, for 4 weeks Procedures: subcutaneous needling, swaying movements and reperfusion approach.	1. Increased clinical efficacy and improved quality of life (*p* < 0.05) 2. Increased CSBMs (*p* < 0.05).	(47)
Post-stroke constipation	Sample size: 40 Age: 61.2 ± 3.79 yrs. Sex ratio: 11:9 Course of disease: 4.31 ± 1.37 yrs.	FSN plus conventional treatment	Frequency: once every other day, with 3 times per week, for 2 weeks Procedure: swaying movements plus 10-s reperfusion approach.	1. Increased total effective rate (87.50% vs. 72.50%) 2. Decreased scores of constipation symptoms (*p* < 0.05) 3. Improved stool form, defecation difficulty, defecation falling feeling, and abdominal distension feeling (*p* < 0.05).	(48)
Pelvic floor dysfunction-related constipation	Sample size: 60 Age: 54.12 ± 3.67 (45–70) yrs. Sex ratio: 17:13 Course of disease: 3.79 ± 1.35 (1–6) yrs.	FSN plus biofeedback therapy	Frequency: 5 times per week for 2 weeks Procedures: swaying movements plus reperfusion approach.	1. Increased total effective rate (93.33% vs. 70.00%) 2. Decreased PAC-QOL and SCL-90 scores (*p* < 0.05) 3. Decreased first rectal sensation and constant rectal sensation (*p* < 0.05) 4. Adverse events, including severe pain, local infection and others, comparable between groups (*p* > 0.05).	(50)
FDD	Sample size: 30 Age: 50.73 ± 14.66 yrs. Sex ratio: 13:17 Course of disease: 68.21 ± 59.20 months. A prospective, randomized trial.	FSN	Frequency: 2 cycles with an interval of 2 days Procedures: swaying movements at a frequency of 100 repeats per minutes plus reperfusion approach	1. Decreased CCS and PAC-QOL scores (*p* < 0.05) 2. Decreased rapid and constant contractions of the pelvic floor (*p* < 0.05) 3. Decreased anal resting pressure, rectal sensation threshold and rectal maximum tolerance, and increased anal maximal contraction pressure (*p* < 0.05) 4. Increased total effective rate (93.3% vs. 79.3% vs. 82.7% in the FSN, electrical stimulation, and electromagnetic stimulation groups, respectively) 5. Decreased recurrent rate (6.6% vs. 37.9% vs. 27.5% in the FSN, electrical stimulation, and electromagnetic stimulation groups, respectively).	(51)

FSN, Fu’s subcutaneous needling; STC, slow transmission constipation; CSBMs, complete spontaneous bowel movements; PAC-QOL, Patient-Assessment of Constipation Quality of Life; BSFS, Bristol Stool Form Scale; SCL-90, Symptom Checklist-90; FC, functional constipation; GQOLI-74, Generic Quality of Life Inventory-74; FDD, functional defecation disorder; CCS, Cleveland constipation score.

Anorectal diseases, such as hemorrhoids, anal fissures, abscesses, fistulas and functional anorectal pain, are common in modern society due to unhealthy life habits, and typically treated with topical medication and surgical approaches. As described in Table 4, FSN brings a convenient, easy-to-go, effective option for individuals bothered by anorectal diseases. Fan-shaped swaying movements of FSN on MTrPs significantly decrease the severity and duration of rectal prolapse (53–55). FSN is also reported to treat benign anal diseases, serving as a promising adjuvant treatment to ease pain and promote the recovery (57, 58). In the future, the underlying mechanism of FSN in treating anorectal diseases needs to be further elucidated.

**Table 4:** A summary of clinical applications of FSN to anorectal diseases.

Disease	Study design	Demographics	Interventions	FSN procedures	Major findings	Ref.
Rectal prolapse	Case report	A 32-year-old male case	FSN	Fan-shaped swaying at a frequency of 90–100 movements per minute plus 3–5 times of reperfusion (relaxation of the right hamstring, and left biceps femoris, and hamstring muscles), for 3–5 repeats	1. Improved surface electromyographic performances of the pelvic floor, right-sided semitendinosus, semimembranosus, and biceps brachii muscle, and left-sided biceps femoris and hamstring muscles.	(55)
Rectal prolapse after PPH for mixed hemorrhoids	RCT	Sample size: 45 Age: 41.85 ± 4.26 yrs. Sex ratio: 26:19 Course of disease: 1.95 ± 0.63 yrs.	FSN plus conventional nursing care	Frequency: once every other day for 6 times Procedures: 40–50 fan-shaped swaying movements per minute plus 3 cycles of 2-min reperfusion approach.	1. Increased overall efficacy (*p* < 0.05) 2. Decreased severity and duration of rectal prolapse (*p* < 0.05) 3. Improved defecation and alleviated perianal edema (*p* < 0.05).	(54)
Rectal prolapse after surgery for mixed hemorrhoids with the syndrome of downward flow of damp-heat	RCT	Sample size: 32 Age: 42.06 ± 2.56 yrs. Sex ratio: 17:14	FSN plus conventional management	Frequency: once every other day for 3 times as a cycle, followed by 2-day off and another cycle; Procedures: 40–50 fan-shaped swaying movements per minute plus3 cycles of 2-min reperfusion approach.	1. Increased overall efficacy (*p* < 0.05) 2. Decreased severity on day 5 and duration of rectal prolapse on day 12 (*p* < 0.05) 3. Pain alleviation on day 5 and 12 (*p* < 0.05) 4. Improved defecation and alleviated perianal edema (*p* < 0.05).	(53)
Postoperative anal hemorrhoids	RCT	Sample size: 36 Age: 40 ± 5 yrs. Sex ratio: 4:5 Course of disease: 2.91 ± 1.23 yrs.	FSN plus anti-infection and TCM herbal fumigation	Frequency: 30 min per time, once daily for 5 times a week, and given for 2 weeks Procedures: rhythmic swaying movements.	1. Decreased scores of anal pain, wound edema and granulation (*p* < 0.05) 2. Increased anal systolic pressure, anal diastolic pressure and anal resting pressure, and decreased anal longest contraction time (*p* < 0.05) 3. Increased serum *β*-EP and VEGF levels (*p* < 0.05) 4. Increased SF-36 scores (*p* < 0.05).	(56)
Benign anal diseases	RCT	Sample size: 80 Age: 48.31 ± 6.88 (30–60) yrs. Sex ratio: 23:17 Course of disease: 6.82 ± 1.32 (3–12) yrs.	FSN	Frequency: once daily for 3 days Procedures: fan-shaped swaying movements at a frequency of 110 repeats per minute on the pain site or the middle of the surgical incision for 20 min with an interval of 2 min, plus reperfusion approach.	1. Decreased VAS scores at 12, 24, 48 and 72 h postoperatively (12 h: 6.32 ± 1.12 vs. 6.89 ± 1.14; 24 h: 5.08 ± 0.85 vs. 5.49 ± 0.88; 48 h: 2.04 ± 0.55 vs. 2.54 ± 0.78; 72 h: 1.23 ± 0.30 vs. 1.98 ± 0.49) 2. Increased overall efficacy rate (97.50% vs. 88.75%) 3. Low incidence of adverse events (3.75% vs. 15.00%).	(57)
Functional anorectal disease	RCT	Sample size: 30 Age: 58.23 ± 9.70 yrs. Sex ratio: 7:23 Course of disease: 18.93 ± 12.33 yrs.	FSN	Frequency: 2–3 times per week for 4 weeks Procedures: swaying movements on the lateral rectus abdominis plus 10-s reperfusion twice.	1. Decreased VAS scores at 2, 4, and 8 weeks (*p* < 0.001) 2. Increased SF-36 scores (*p* < 0.001) 3. Decreased SDS and SAS scores (*p* < 0.001) 4. Increased overall efficacy (83.3% vs. 70.0%) and cure rate (13.3% vs. 6.7%).	(58)

FSN, Fu’s subcutaneous needling; RCT, randomized controlled trial; PPH, procedure for prolapse and hemorrhoids; TCM, traditional Chinese medicine; SF-36; 36-Item Short-Form Survey; VAS, Visual Analogue Scale; SDS, Self-Rating Depression Scale; SAS, Self-Rating Anxiety Scale.

Though FSN is a highly safe approach to manage pain and non-pain conditions, minimal adverse events are reported in the treatment of constipation. Wu et al. showed that the incidence of pain and local infection is comparable between the FSN + biofeedback group and the biofeedback group (10.00% vs. 8.33%), suggesting that FSN does not additionally cause adverse events during the treatment of pelvic floor arrhythmia constipation (53). Yuan et al. (52) reported mild adverse events in the treatment of functional constipation with FSN plus Bifidobacterium triple viable capsules, including 2 cases of headache and dizziness, 1 case of frequent micturition, and 2 cases of nausea and vomiting. However, the overall incidence of complications is significantly lower in constipation patients treated with FSN + Bifidobacterium triple viable capsules than those treated with Bifidobacterium triple viable capsules alone (7.84% vs. 28.30%). Overall, we suggested that FSN is characterized as the high safety in alleviating lower gastrointestinal disorders like constipation.

## 4 Outlook and future development

### 4.1 Expanding the application of FSN to gastrointestinal and other disorders

FSN evolves from dry needling to control an expanding range of pain-related disorders, such as musculoskeletal, neuralgia and visceral pain, or even gastrointestinal disorders. The bidirectional gut-brain axis links the central nervous system (CNS) with gastrointestinal tract. It effectively connects the CNS, enteric nervous system (ENS) and autonomic nervous system (ANS). Generated in the brain, regulatory signals are transmitted to the ENS through the autonomic nervous system or neuroendocrine system. This delicate and complex process can be mediated via FSN in the subcutaneous layer on MTrPs. FSN mediates the gut-brain axis by directly altering expression levels of excitory (e.g., motilin, gastrin, substance P, ghrelin, 5-HT) and inhibitory neurotransmitters (e.g., vasoactive intestinal peptide, cholecystokinin, somatostatin, neurotensin, nitric oxide), thereafter alleviating gastrointestinal and anorectal diseases. In the future, the molecular mechanisms by which FSN alleviates lower gastrointestinal disorders require to be demystified.

### 4.2 Deepening the theoretical system of FSN

While the tightened muscle and reperfusion approach are the two cornerstones supporting the therapeutic mechanisms of FSN, a box of issues requires further in-depth exploration. First of all, a standardized and objective approach is needed to identify tightened muscles and MTrPs in specific conditions. Modern imaging techniques, biomechanical analysis, and surface electromyography are expected to assist in the precise recognition of tightened muscles. Second, the exact molecular mechanisms and signal transduction underlying the reperfusion approach of FSN in masking pain and recently, non-pain conditions, have not been fully elucidated. In the future, studies should be performed to evaluate the combination of FSN with pharmacological treatment, traditional needling, physical therapy and other treatment strategies. A deep mining of the theoretical system of FSN offers scientific support to its expanded clinical practices.

### 4.3 Technical innovation

Technical innovations are expected to transform the treatment with FSN. Newly designed FSN needles and procedures are not surprisingly bringing benefits to more individuals. For example, FSN needles designed by materials with a high biocompatibility or equipped with different elastic moduli are applicable to subcutaneous tissues at distinct sites without causing allergic reactions. Intelligent modules embedded in FSN needles, such as built-in sensors, can catch the data about tissue resistance, temperature and other parameters in real time, thus providing more accurate feedback information.

### 4.4 Risk of bias in the current FSN research

Evidence-based medical research can widen the recognition of FSN. Currently, clinical trials of FSN in treating pain and non-pain conditions are scant. Most of them are limited by a small sample size, restrictions in only Chinese people, and lack of recognized standardized control approaches. Multi-center studies are essential for validating the efficacy and safety of FSN across regions and populations. In addition, long-term follow-up studies, real-world analysis, cohort studies, and randomized controlled trials targeting FSN are urgently needed to offer evidence for the efficacy of FSN.

## 5 Conclusion

FSN has achieve favorable outcomes in alleviating pain and non-pain conditions, especially in treating lower gastrointestinal and/or anorectal diseases. More clinical and animal trials are essential for proving the efficacy and safety of FSN. New medical technology will transform FSN into a more beneficial treatment modality.

## References

[1] Park J Oh J Bak S Yun S Kim C Chu H . Fu’s subcutaneous needling: focusing on clinical usage and treatment protocol. J Korean Med Soc Soft Tissue. (2021) 5:60–8. doi: 10.54461/JKMST.2021.5.1.60

[2] Fu ZH Wang JH Sun JH Chen XY Xu JG. Fu's subcutaneous needling: possible clinical evidence of the subcutaneous connective tissue in acupuncture. J Altern Complement Med. (2007) 13:47–52. doi: 10.1089/acm.2006.6125, 17309377

[3] Chiu PE Wu CC Lu DJ Fu Z Chou LW. Effectiveness of Fu's subcutaneous needling in pain management and rehabilitation: a narrative review. Rehabil Pract Sci. (2025) 2025:14. doi: 10.6315/3005-3846.2261

[4] Wu F. Zhang W. Gan X. Guan J. Song Y. Mao M. Fu's subcutaneous needling: a novel acupuncture treatment for musculoskeletal pain diseases. Acupuncture - Resolving Old Controversies and Pointing New Pathways. Marcelo S Roberta de Medeiros London, UK: IntechOpen. (2019) 28. doi: 10.5772/intechopen.84251,

[5] Fu Z Lu D. "Fu’s subcutaneous needling: a novel therapeutic proposal". In: Acupuncture - Resolving Old Controversies and Pointing New Pathways. Marcelo S Roberta de Medeiros, eds. London, UK: IntechOpen (2019). p. 21.

[6] Fu ZH Chen XY Lu LJ Lin J Xu JG. Immediate effect of Fu's subcutaneous needling for low back pain. Chin Med J. (2006) 119:953–7. 16780777. doi: 10.1097/00029330-200606010-00014, 16780777

[7] Huang CH Lin CY Sun MF Fu Z Chou LW. Efficacy of Fu’s subcutaneous needling on myofascial trigger points for lateral epicondylalgia: a randomized control trial. Evid Based Complement Alternat Med. (2022) 2022:1–10. doi: 10.1155/2022/5951327, 35321501 PMC8938053

[8] Lu D Sun J Xu N Chou LW. Benefits of Fu’s subcutaneous needling treatment for isolated oculomotor nerve paralysis after traumatic brain injury: a case report. QJM. (2024) 117:882–4. doi: 10.1093/qjmed/hcae174, 39213312

[9] Tao L Li J Lu J Zhu M Xie Z Bao X . Fu's subcutaneous needling for non-acute idiopathic facial paralysis: a randomized controlled trial. Chinese Acupunct Moxib. (2024) 44:1249–53. doi: 10.13703/j.0255-2930.20240611-k0001, 39532440

[10] Peng B Wu W Hou S Li P Zhang C Yang Y. The pathogenesis of discogenic low back pain. J Bone Joint Surg Br. (2005) 87-B:62–7. 15686239. doi: 10.1302/0301-620X.87B1.15708, 15686239

[11] Biyani A Andersson GBJ. Low back pain: pathophysiology and management. J Am Acad Orthop Surg. (2004) 12:106–15. doi: 10.5435/00124635-200403000-00006, 15089084

[12] Ma KL Zhao P Cao CF Luan FJ Liao J Wang QB . Fu's subcutaneous needling versus massage for chronic non-specific low-back pain: a randomized controlled clinical trial. Ann Palliat Med. (2021) 10:11785–97. doi: 10.21037/apm-21-2986, 34872303

[13] Zhang DQ Fu ZH Sun J Song YJ Chiu PE Chou LW. Effects of Fu's subcutaneous needling on clinical efficacy and psychological cognitive characteristics in patients with chronic non-specific low back pain: a randomized controlled trial. Complement Ther Med. (2024) 85:103080. doi: 10.1016/j.ctim.2024.103080, 39214379

[14] Li H Yang CC Bai T Sun J Fu Z Mi J . The impact of Fu’s subcutaneous needling on lower limb muscle stiffness in knee osteoarthritis patients: study protocol for a pilot randomized controlled trial. J Pain Res. (2024) 17:3315–26. doi: 10.2147/JPR.S482082, 39403099 PMC11471907

[15] Mou JJ Wang Q Wu J Zhang LX Li YA Luo ZC . The effect of Fu's subcutaneous needling in treating knee osteoarthritis patients: a randomized controlled trial. Explore. (2024) 20:562–71. doi: 10.1016/j.explore.2023.12.015, 38176976

[16] Chiu PE Fu ZH Sun J Jian GW Li TM Chou LW. Efficacy of Fu's subcutaneous needling in treating soft tissue pain of knee osteoarthritis: a randomized clinical trial. J Clin Med. (2022) 11:7184. doi: 10.3390/jcm11237184, 36498758 PMC9740707

[17] Chiu PE Fu ZH Sun J Jian GW Li TM Chou LW. Fu's subcutaneous needling for knee osteoarthritis pain. J Vis Exp. (2023) 24:193. doi: 10.3791/65299, 37036213

[18] Yang X Wang H Sun J. Understanding tightened muscle in knee osteoarthritis and the impacts of Fu's subcutaneous needling: a pilot trial with shear-wave elastography and near-infrared spectroscopy. Medicine (Baltimore). (2024) 103:e38274. doi: 10.1097/MD.0000000000038274, 38787967 PMC11124628

[19] Liu Z Ma L Bi HY. Clinical effect on knee osteoarthritis treated with Fu's subcutaneous needling therapy. World J Acupunct Moxibustion. (2020) 30:29–32. doi: 10.1016/j.wjam.2020.02.001

[20] Zhang YH Wu T Shen W Huang FY Liu CR. Effect of Fu's subcutaneous needling on thickness and elasticity of affected muscles in shoulder neck pain based on ultrasonic elastography. Zhongguo Zhen Jiu. (2020) 40:939–41. doi: 10.13703/j.0255-2930.20190817-k0001, 32959587

[21] Bao X Wang MH Liu H Shi HF Salem Y Xu S . Treatment effect and mechanism of Fu's subcutaneous needling among patients with shoulder pain: a retrospective pilot study. Anat Rec. (2021) 304:2552–8. doi: 10.1002/ar.24710, 34324795

[22] Zhen Y. Fu's subcutnaeous needling combined with manipulation in treating 120 cases of neck and shoulder pain. People's Mil Surg. (2003) 46:555–5. doi: 10.3969/j.issn.1000-9736.2003.09.043

[23] Navarro-Ledesma S Hamed-Hamed D Gonzalez-Muñoz A Pruimboom L. Impact of physical therapy techniques and common interventions on sleep quality in patients with chronic pain: a systematic review. Sleep Med Rev. (2024) 76:101937. doi: 10.1016/j.smrv.2024.101937, 38669729

[24] Wu CY Chou LW Huang SW Liao WL Chang SM Lee HC . Effects of Fu’s subcutaneous needling on postoperative pain in patients receiving surgery for degenerative lumbar spinal disorders: a single-blind, randomized controlled trial. J Pain Res. (2024) 17:2325–39. doi: 10.2147/JPR.S465417, 38974828 PMC11227350

[25] Feng J Zhang L Pan XF. Fu's subcutaneous needling on the pain point influences the efficacy, pain and pain-causing factors of elderly patients after osteoporotic vertebral compression fracture. J Sichuan Tradit Chin Med. (2024) 42:205–7. doi: 10.3969/j.issn.1000-3649.2024.3.sczy202403056

[26] Cheng A Li XL Tian J. Clinical application of Fu's subcutaneous needling to early analgesia after lumbar compression fracture treated with PVP surgery. Health Med Res Pract. (2024) 21:81–4. doi: 10.11986/j.issn.1673-873X.2024.S2.16

[27] Long C Shuchuan Z Xin S Qiang M Xi F Jing W . Observation of the therapeutic effect of Fu's subcutaneous needling on residual lower back pain in elderly patients after PVP surgery (2025) 6:1–7. doi: 10.25236/AJMHS.2025.060301

[28] Guo LH Chen X Huang P Liang YC Mu JP. Clinical study on Fu’s subcutaneous needling with laser for postherpetic neuralgia. J Acupunct Tuina Sci. (2014) 12:165–8. doi: 10.1007/s11726-014-0766-x

[29] Han WH Liu WA Ren Z Qiu L Yan TY Zhu MH. Clinical thinking on the treatment of postherpetic neuralgia with Fu's subcutaneous needling. Yunnan J Tradit Chin Med Mater Med. (2025) 46:81–5. doi: 10.3969/j.issn.1007-2349.2025.04.018

[30] Gao Y Sun J Fu Z Chiu PE Chou LW. Treatment of postsurgical trigeminal neuralgia with Fu’s subcutaneous needling therapy resulted in prompt complete relief: two case reports. Medicine (Baltimore). (2023) 102:e33126. doi: 10.1097/MD.0000000000033126, 36862912 PMC9981408

[31] Li Y Gao X Huang H Zhou X Zang Y Chou LW. Effects of Fu’s subcutaneous needling on mitochondrial structure and function in rats with sciatica. Mol Pain. (2022) 18:17448069221108717. doi: 10.1177/17448069221108717, 35670088 PMC9210095

[32] Snijders RAH Brom L Theunissen M denBeuken-van Everdingen v. Update on prevalence of pain in patients with cancer 2022: a systematic literature review and meta-analysis. Cancers (Basel). (2023) 15:591. doi: 10.3390/cancers15030591, 36765547 PMC9913127

[33] Chen JP Chen N Tan HS He JY. Effects of Fu’s subcutaneous needling combined with oxycodone hydrochloride sustained release tablets on inflammatory factors and pain in patients with moderate and severe cancer pain. Med Health Res. (2024) 8:117–9. doi: 10.3969/j.issn.2096-3718.2024.18.038

[34] Zhong H. Observation on the efficacy of Fu’s subcutaneous needling assisted in the treatment of moderate cancer pain. Chin J Diag. (2016) 5:148–51. doi: 10.3877/cma.j.issn.2095-3240.2016.04.005

[35] Liu XL Liu ZX Cai XM He YH Li JL Liu YY. Clinical observation of the impact of Fu’s subcutaneous needling on the quality of life of patients with cancer pain. Shenzhen J Integr Tradit Chin Western Med. (2022) 32:28–31. doi: 10.16458/j.cnki.1007-0893.2022.16.009

[36] Li Q Tian FL Guo ZY Zhou LS Zhu HS. Effect of Fu’s subcutaneous needling on visceral sensitivity, gastrointestinal motility, gut microbiota and intestinal mucosal barrier function in patients with mild and moderate diarrhea syndrome. Chin Gen Pract. (2021) 24:1111–1115+1130. doi: 10.12114/j.issn.1007-9572.2020.00.608

[37] Li XQ Chen R Chen MX Zheng XH. Clinical observation of Fu’s subcutaneous needling combined with Western medicine for the treatment of irritable bowel syndrome. J Guangzhou University Tradit Chin Med. (2024) 41:122–8. doi: 10.13359/j.cnki.gzxbtcm.2024.01.019

[38] Ni HH. Research on the effect of Fu’s subcutaneous needling on hypersensitivity of internal organs in patients with diarrhea irritable bowel syndrome. Nanjing University Chin Med. (2024). doi: 10.27253/d.cnki.gnjzu.2024.000046

[39] Qiu YM Wang ZY Shuai YC. Fu’s subcutaneous needling combined with umbilical moxibustion on the umbilical irritable bowel syndrome and its effect on the gastrointestinal hormone level and gut microbiota composition. Shaanxi J Tradit Chin Med. (2023) 44:250–4. doi: 10.3969/j.issn.1000-7369.2023.02.026

[40] Tu YQ. Observation on the Clinical Efficacy of Fu’s Subcutaneous Needling Combined with Wumei Pills for the Treatment of Diarrhea-Type Irritable Bowel Syndrome (Mixed Syndrome of Cold and Heat). Nanchang: Jiangxi University Chin Med (2024).

[41] Chen H Sun F Li H Sun HJ. Clinical observation of Fu’s subcutaneous needling combined with self-designed prescription for the treatment of chronic functional constipation. China's Naturopathy. (2021) 29:83–6. doi: 10.19621/j.cnki.11-3555/r.2021.0832

[42] Jiang YX Liu JH Liu X Ji EF Liu SJ Yang HJ. Jiawei Zhishu decoction combined with Fu’s subcutaneous needling for the treatment of slow-transmitting constipation and spleen deficiency and qi stagnation. Acta Chin Med. (2025) 40:661–7. doi: 10.16368/j.issn.1674-8999.2025.03.108

[43] Li G Xu BY Cai YY Ding HL Wang L Hu YJ . A comparative study on the effect of Fu’s subcutaneous needling and filiform needling combined with biofeedback on colon dynamics and serum cerebral intestinal peptides in patients with slow transmission constipation. Mod Digestion Interv. (2024) 29:1085–9. doi: 10.3969/j.issn.1672-2159.2024.09.011

[44] Li G Xu BY Cai YY Ding HL Wang L Zhang HY . Clinical observation of Fu’s subcutaneous needling combined with biofeedback for the treatment of qi stagnation and slow transmission constipation. J Sichuan Tradit Chin Med. (2024) 42:197–9. doi: 10.3969/j.issn.1000-3649.2024.11.sczy202411060

[45] Liu WL Han HW Wei FM. Clinical observation of Fu’s subcutaneous needling combined with pelvic floor biofeedback therapy for the treatment of functional constipation. China Naturopathy. (2022) 30:52–5. doi: 10.19621/j.cnki.11-3555/r.2022.0818

[46] Ni HH. Effect of Fu’s subcutaneous needling on the symptom score of patients with functional constipation. Nei Mongol J Tradit Chin Med. (2021) 40:123–4. doi: 10.16040/j.cnki.cn15-1101.2021.01.074

[47] Niu P Wu QW Feng JZ Lin YL. Observation on the effect of Fu’s subcutaneous needling combined with rhubarb powder acupoint patching for the treatment of constipation after stroke. Chin Med Pharm. (2024) 14:115–8. doi: 10.20116/j.issn2095-0616.2024.05.26

[48] Qiu B. Clinical Observation of the Treatment of Constipation after Stroke with Fu’s Subcutaneous Needling Combined with Reperfusion Exercise. Hangzhou: Zhejiang Chinese Medical University (2020).

[49] Tan Y Yuan LJ Fu ZH. The clinical effect of Fu’s subcutaneous needling in the treatment of functional constipation. Chin Med Herald. (2019) 16:155–8.

[50] Wu CH Chen R Li H Chen Y Xu S. Clinical study on the treatment of pelvic floor arrhythmia constipation with biofeedback combined with Fu’s subcutaneous needling. Nei Mongol J Tradit Chin Med. (2023) 42:84–6. doi: 10.16040/j.cnki.cn15-1101.2023.04.081

[51] Yan S Wang XP Le YZ Liu Y Liu J Pan L . Fu’s subcutaneous needling for functional bowel movement disorders. Acta Chin Med. (2025) 40:1569–76. doi: 10.16368/j.issn.1674-8999.2025.07.253

[52] Yuan JL Shi ZR Zhai ZZ Song JG Li ML. Observation on the efficacy of Fu’s subcutaneous needling combined with Bifidobacterium triple viable capsules in the treatment of functional constipation. Mod J Integr Tradit Chin West Med. (2024) 33:829–32. doi: 10.3969/j.issn.1008-8849.2024.06.020

[53] Chen B. Clinical Study of Fu’s Subcutaneous Needling in Treating anal Prolapse after Surgery for damp-heat-Beating Mixed Hemorrhoid. Changsha: Hunan University of Chinese Medicine (2022).

[54] Sun BB. Clinical observation of Fu’s subcutaneous needling on anal swelling after PPH surgery for mixed hemorrhoid. Chin Health Care. (2023) 41:15–22. doi: 10.88888/j.1009-8011.2023.22.15-17

[55] Wang TL Zhang P Lu WW Gao Y Fang QY Huang YE. Observation of the immediate efficacy of Fu’s subcutaneous needling in the treatment of anal swelling based on biofeedback surface electromyography evaluation: a case report. Chin J Coloproctol. (2025) 45:74–6.

[56] Wang XL Yang XY Dong SH Li MW. Effects of Fu’s subcutaneous needling combined with Chinese medicine fumigation on pain, wound healing, quality of life, and serum β-EP and VEGF levels in patients after hemorrhoid surgery. Shanghai J Acupunct Moxibust. (2023) 42:737–42. doi: 10.13460/j.issn.1005-0957.2023.07.0737

[57] Xie YM Lan B Chen MH. The application effect of Fu’s subcutaneous needling on postoperative pain of benign anal diseases. Chin Health Stand Manag. (2025) 16:140–3. doi: 10.3969/j.issn.1674-9316.2025.01.034

[58] Zhang CX. Observation on the Efficacy of Fu’s Subcutaneous Needling in the Treatment of Functional Anorectal pain. Guangzhou: Guangzhou University of Chinese Medicine (2024).

